# Distributed Blockchain-Based Platform for Unmanned Aerial Vehicles

**DOI:** 10.1155/2022/4723124

**Published:** 2022-08-31

**Authors:** Tariq Ahamed Ahanger, Abdulaziz Aldaej, Mohammed Atiquzzaman, Imdad Ullah, Muhammad Yousufudin

**Affiliations:** ^1^College of Computer Engineering and Sciences, Prince Sattam Bin Abdulaziz University, Al-Kharj, Saudi Arabia; ^2^School of Computer Science, University of Oklahoma, Norman, OK, USA

## Abstract

Internet of Things (IoT)-inspired drone environment is having a greater influence on daily lives in the form of drone-based smart electricity monitoring, traffic routing, and personal healthcare. However, communication between drones and ground control systems must be protected to avoid potential vulnerabilities and improve coordination among scattered UAVs in the IoT context. In the current paper, a distributed UAV scheme is proposed that uses blockchain technology and a network topology similar to the IoT and cloud server to secure communications during data collection and transmission and reduce the likelihood of attack by maliciously manipulated UAVs. As an alternative to relying on a traditional blockchain approach, a unique, safe, and lightweight blockchain architecture is proposed that reduces computing and storage requirements while keeping privacy and security advantages. In addition, a unique reputation-based consensus protocol is built to assure the dependability of the decentralized network. Numerous types of transactions are established to characterize diverse data access. To validate the presented blockchain-based distributed system, performance evaluations are conducted to estimate the statistical effectiveness in the form of temporal delay, packet flow efficacy, precision, specificity, sensitivity, and security efficiency.

## 1. Introduction

Different industries use unmanned aerial vehicles (UAVs) for civilian, military, commercial, and governmental sectors [1]. There are several examples of environmental monitoring in the nonmilitary sector (such as pollution, plant health, and industrial accidents). After a natural catastrophe or a terrorist attack or other emergency situation, the military and government often use surveillance and delivery technologies to collect or convey data and/or distribute supplies. Products and supplies can be delivered in metropolitan regions as well as rural ones. Remote control and monitoring are made possible by UAVs' reliance on sensors, antennae, and embedded software, which makes them an integral element of the Internet of Things (IoT) [2].Additionally, UAVs will be able to monitor essential infrastructure, such as power lines, when battery and quick charging technology improves. Using UAVs to monitor the atmosphere from 2 to 3 kilometers above the ground is another applicability illustration. Weather forecasting algorithms can benefit from the pooled data, which includes temperature, wind and turbulence, and airspeed [3]. Using UAVs may lead to exposure to a variety of cyberattacks, including the Sybil attack, DoS/DDoS attack, and GPS spoofing. It could lead to the destruction of the entire system's data availability if the untrusted communication channel is breached [4]. As a result, device authentication and communication security are a major concerns. A central server or cloud or fog computing is used to process and store data in the UAV system. Because of the inherent flaws of conventional centralized design and cloud server manipulation, the integrity of data may be compromised when a server is hacked, which is also a concern with the conventional centralized architecture [5].

### 1.1. Research Domain

**Figure 1:**
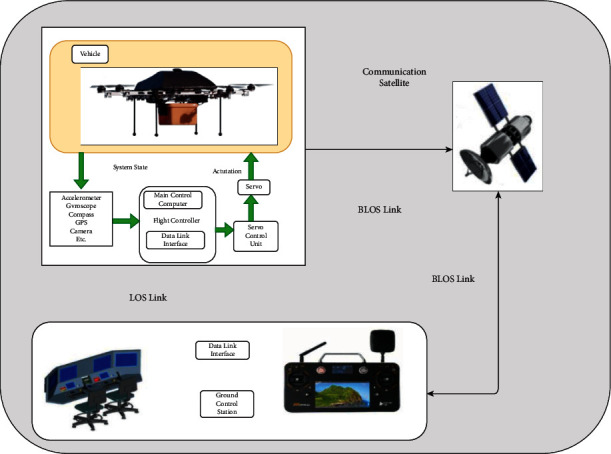
Conceptual overview of UAV architecture.

**Figure 2:**
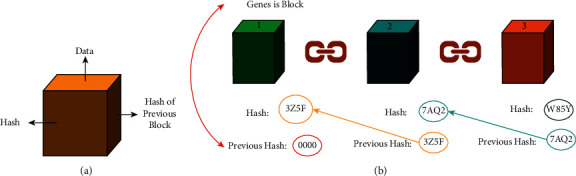
Illustration of blockchain architecture; (a) single block; (b) blockchain chain formulation.

UAV technology is seeing a growing trend of autonomy, driven by developments in batteries, charging techniques, and embedded software that uses machine learning algorithms to learn [6]. It is being created and tested; however, there are currently no permitted autonomous UAV systems that may be used. Semiautonomous UAVs clearly show that a fleet of UAVs with advanced algorithms may be capable of handling a variety of human-defined tasks and developing issues with high levels of coordination by ensuring contact between UAVs and ground control station (GCS) [7].  Figure 1 shows the conceptual overview of the conventional UAV architecture (Source: http://www.dronefromchina.com/new/drone-communicate-with-UAV-ground-stations.html). Military planes have long used the term “*a swarm of UAVs*” to describe a large group of drones. Conspicuously, future UAVs will need a secure and private network system, as well as an integrated system that is responsive and sustainable to develop a trusted, integrated environment [8]. A huge number of linked devices, transaction processing, and coordination between many devices in the context of IoT may be achieved using blockchain (BC) technology, which is the rudimentary mechanism of Ethereum along with other cryptocurrencies [9]. Aside from being a tamper-proof, and immutable recording of data in a network (i.e., a log), BC is also a decentralized architecture that prevents a single point of failure, making it a more resilient and stable platform for IoT execution.  Figure 2 shows the illustration of the blockchain (Source: https://github.com/rajibdpi/BlockChain). The underlying cryptographic algorithm employed by BC, including hash functions, symmetric encryption, and digital signatures, is a key component of BC [10]. Many nonmonetary situations for IoT security have already been used in BC, such as the protection of health care data, government democracy and legal enforcement, smart home, smart toy at the edge computing, and vehicle-to-infrastructure communication system [11, 12]. It is difficult to integrate blockchain technology with IoT because of the high resource consumption, high delays, and high memory overhead to store the records of billions of transactions [13]. When it comes to research on the integration of BC with UAVs, most of the studies focus on employing BC technology as a decentralized database [14].

A distributed blockchain-based architecture for UAVs is proposed in the current research to overcome the aforementioned security and privacy issues while also providing a high degree of operational autonomy. The following are the most significant contributions:

1. UAV applications are explored for vulnerability detection in the IoT domain for data security and protection.
2. The blockchain framework is designed to alleviate the demand for storage and calculation placed on each UAV by employing novel transaction and block structures over lightweight cryptography.
3. A novel consensus mechanism analogous to Delegated Proof of Stake (DPoS) paired with a reputation assessment system is implemented to achieve an agreement with the aggregated data amongst UAVs.
4. The blockchain-based decentralized architecture is validated for UAVs in which numerous tests are conducted to determine self-defense capabilities.

#### 1.1.1. Paper Structure

Section 2 provides an overview of some of the important contributions in the related domain of study. Introduction to the UAV networks and BC technology is provided in Section 3. Block and transaction structures are shown in Section 4 to explain the system architecture required for the proposed semiautonomous BC-based UAV framework.  Section 5 explains how the proposed framework for UAVs works. A security assessment and an efficiency assessment are included in the performance analysis presented in Section 6.  Section 7 presents some of the open issues and challenges for future research. Finally, Section 8 concludes the paper.

## 2. Literature Review

### 2.1. Internet of Things (IoT) Security

Kanuparthi et al. [15] depicted that as the number of smart devices in IoT rises, the risk of cyber-attacks including buffer overflow attacks is also elevated. If the device is hacked, it might lead to a data leak and expose the server to attacks. Authors have incorporated hardware security primitives to address the security aspect. However, the presented technique takes a lot of computing power from the devices. According to Misra et al. [16], IoT devices did not evaluate security needs carefully; therefore, it can lead to enormous cyberattacks. For better privacy, several attempts are being made to analyze the security of UAV communication networks. Rudinskas et al. [17] examined the radio communication system and discussed radio-related challenges, harmful threats, and potential solutions for data transformation between various entities. The presented study focused on cryptography approaches while ignoring the limitations of UAVs in terms of space and power. To assist researchers and end-users in better grasping the system's risk profile, Thing et al. [18] provided a clear and comprehensive security model for UAVs. Many UAV-related studies have focused on resisting GPS spoofing and jamming attempts due to the system's architecture as a GPS location system. A taxonomy of UAV cyberattacks and future research directions were provided by Krishna et al. [19] based on an in-depth examination of numerous GPS spoofing and jamming attacks. GPS spoofing attacks were researched in detail, including their design and impact. A new detection and mitigation approach was presented and tested on the most promising test platform. According to the technique presented by Javaid et al. [2], spoofing is caused by the lack of encryption of GPS information. MP-OLSR, the multi-path routing protocol introduced by Randu et al. [20] for FANET, aggregates dynamic data with high mobility in emergency scenarios. There is no discussion of a malicious node scenario for the technique in the simulation platform. A multilayer security framework for WiFi-based UAV systems was established by Hooper et al. [11]. Authors demonstrated how the proposed method mitigated three adversarial attacks: *Buffer-overflow attack*, *DoS attack*, and *ARP cache poison attack*. A different study by Zhang et al. [21] found that by simply relocating the UAV, the legal link could be created strongly than the eavesdropping link and that it may be used to combat eavesdropping in UAV-to-ground (U2G) and ground-to-UAV (G2U) communications.

### 2.2. Blockchain Security for IoT

The unchangeable and distributed ledger of the blockchain has drawn the interest of many academics [31]. However, the blockchain business has faced several difficulties as a result of the widespread use of IoT devices [32]. Tosh et al. [33] presented that consensus procedures in blockchain are a serious issue that might lead to a delay in the consensus of proof of work. Using a combination of private and public ledgers on local networks, a blockchain topology suitable for smart homes has been proposed by Dorri et al. [34]. Smart gadgets with edge computation and vehicle-to-infrastructure communication schemes were investigated by Yang et al. [35]. Numerous studies have been carried out to examine the success of integrating blockchain technology with UAV systems in many areas as blockchain may provide many benefits to various IoT situations. Using private blockchain to distribute and store group keys, as well as handle the dynamic list of network members, Li et al. [36] suggested a mutual-healing group key distribution technique. Recovering an unmanned aerial vehicle (UAV) that has crashed is challenging; thus, Scarlato et al. [37] devised an authorized side chain with proof of authority consensus recording GPS coordinates and flying altitude for the avoidance and recovery of the wrecked UAVs. In an air-to-ground IoT network, Zhu et al. [38] studied trading and storage management difficulties and presented a novel consensus method based on Nash equilibrium to minimize resource usage. UAVs have limited processing resources; therefore, Kuzmin and Znak [39] introduced a unique proof-of-graph consensus mechanism for an autonomously running UAV network on the blockchain. To ensure data integrity, traceability, and unforgeability, Youssef et al. [40] developed a distributed payment system based on the blockchain between a UAV cloud and a sensor cloud. Rana et al. [41] focused on securing data sent and received between UAVs and the cloud, where GPS data are necessary to be included. Aslam and Shin [42] developed an effective method to verify user identity and identify malicious UAVs by employing a memory-efficient data structure known as the *π*-hash bloom filter. Based on the comprehensive overview, Table 1 has been formulated to depict the comparative analysis with the current work.

## 3. Fundamental Aspects

### 3.1. UAV Communication

UAV systems typically consist of 3 components: *a single UAV* (*or fleet of them*), *ground control stations* (*GCS*), and 3 different types of *data transmission lines* (satellite link, UAV-to-UAV link, and radio link) [43]. Each type of link has a particular purpose. GPS and meteorological data are transmitted between satellite and UAV through satellite connection, while the UAV-UAV link conveys messages of interaction between the two types of UAVs [2]. Finally, a radio communication link delivers GCS orders, audio/video, and other data to the UAVs [44]. In addition, a specific UAV is examined in the current research that has a flight controller, a cluster of sensors, and a set of actuators when discussing the building blocks. Moreover, the internal workings of a UAV are discussed [3]. In other words, UAV is piloted by a flight controller, which serves as a central processing unit for data acquired by different sensors and sent to the control units or relayed to GCS, depending on the mode of control [45]. Other acting actuators are influenced by GCS orders, which are regulated by the flight controller [19]. Drone photography, autonomous freight transport, precise crop monitoring, building surveillance, tracking unsafe circumstances, or providing essentials for emergency services are some of the uses for drones that have been documented in several studies [46]. UAVs exemplify how a multiscale technological ecosystem, such as WiFi, Zigbee, 4G/5G wireless cellular communications, and machine-to-machine (M2M) communication, as well as ancillary computational resources combine to form an embedded system (such as cloud computing and edge computing platform) [47]. Because cybersecurity was not prioritized in the early stages of design, modern UAV autopilot systems are vulnerable to a variety of cyberattacks [37].

### 3.2. UAV Threats

Three hostile cyberattacks aimed at attacking the distributed UAVs system are briefly discussed.

1. *Sybil Attack* peer-2-peer networks are vulnerable to Sybil attack, in which a hacker uses stolen or manufactured identities to represent numerous separate nodes in the network. There are a variety of harmful behaviors that the adversary might use to gain a disproportionate amount of control over data integrity, resource consumption, and overall network performance.
2. *DDoS (denial-of-service) attack* some requests may be prevented from being answered by making requests for the target device to become unavailable.
3. *GPS Spoofing* armed forces use encryption to ensure that GPS signals cannot be tampered with, while civilian GPS transmissions lack both encryption and authentication, making it possible for an adversary to produce or fake the original signals. As a result, the attacker can guide the UAV to a chosen destination that is different from the current intended course by manipulating the signals.

### 3.3. Blockchain Fundamentals

#### 3.3.1. Overview

Although blockchain has been around for a few years now as Nakamato et al. [48] coined the term in 2008. It is been widely regarded as an emerging technology for distributed and decentralized data sharing. Blockchain was initially designed to record money transactions, where each transaction is recorded and saved by all members of the peer-to-peer network, but it is increasingly being used in nonmonetary applications as well [49]. An important feature of blockchain is its ability to demonstrate that an uncentralized group of users may form an agreement that can be recorded in a verifiable and safe manner [42].

#### 3.3.2. Structure of the Blockchain

As shown in Figure 2, the chain is made up of blocks linked together by the hash value of the preceding block. To solve the complicated mathematical problem known as hash functions (i.e. hash functions), a nonce (target value) must be provided for each block in a sequence of transactions recorded in the current block [6]. The *proof of work* concept was developed for Ethereum to make block production computationally “hard” based on standard hash functions, thereby preventing the attackers from tampering with block information. For instance in Ethereum, only miners are responsible for creating blocks and broadcasting newly created blocks back to the blockchain. Once the newly created block has been validated by all parties, it should be added to the blockchain and validated along with the transactions it contains.

## 4. Distributed BC-Based UAVs Framework

Distributed UAV framework based on blockchain technology works is presented in the current framework. Moreover, a customized blockchain structure for UAV communication systems is proposed for secure data transmission.

### 4.1. Overview

**Figure 3:**
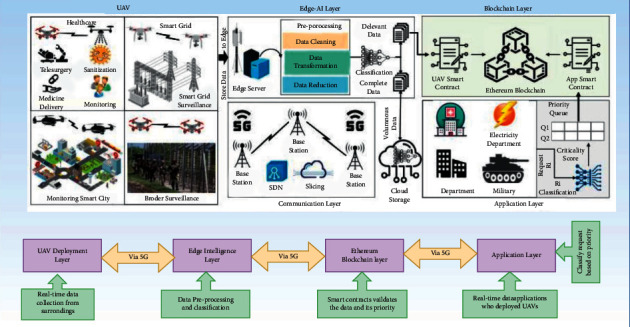
Blockchain-based UAVs framework: workflow analysis.

Figure 3 shows a situation where healthcare surveillance UAVs are used to locate vulnerable patients to control disease outspread. Assuming the UAVs can identify the target individual with the use of identification, including facial recognition, it is assumed that any person walking outdoors in a region may be monitored by numerous surveillance UAVs. Ground control stations (GCS) are task control centers located on land that is responsible for managing UAVs to collect and process large amounts of data. In addition, the cloud server serves different purposes. Data from sensors, pictures, and videos, as well as the status and position of each UAV, may all be saved in the cloud as part of a single storage service. The cloud can handle a wide range of computation-intensive jobs due to the widespread use of high-quality computational tools. For example, UAVs may access the internet and obtain information on no-fly zones. A cloud server (either GCS or UAVs) must provide the hash value of the requested data to the requester.

### 4.2. Blockchain Construction

Expecting UAVs to have the same processing capacity as Ethereum miners makes the challenge of enabling distributed storage and security rather difficult for the IoT device-based network. As a result of these findings, a customized blockchain architecture is depicted in the suggested framework.

### 4.3. Block-Level Information

**Figure 4:**
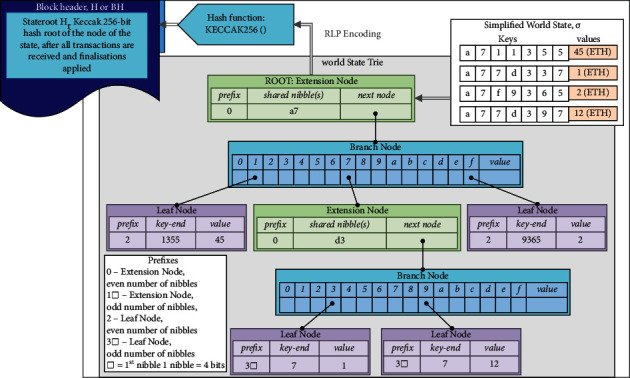
Example of the modified Merkle Patricia tree.

Reformatory blocks, like Ethereum's, may be broken down into 2 parts: the block header and the block body. The proposed block is tailored to the needs of lightweight IoT devices and UAV communications by employing lightweight cryptography technologies such as Keccak (i.e., a low-cost alternative to the standard version that was selected as the winner of SHA-3 by NIST) and redefining the functions of all transactions. Block headers are made up of the current block header's hash, the previous block header's hash, the root of a reputation tree, a policy list, and timestamp as shown in Table 2. Evaluation of reputation scheme is performed similarly to the fundamental idea of Delegated Proof of Stake (DPoS) to nominate a node to generate a new block, unlike Ethereum where the miners must find a solution to a hash puzzle to win the right of append to the main chain the new block. As a result, the reputation tree item is included in the block, and the block header records the tree's root. When adding new UAVs to the system during start-up, the GCS administrator generates a policy list, which is then included in Block 1. Each node in the network should refer to the most recent policy to process transactions because updating it is as simple as making a change to the policy list in the most recent block. A reputation tree and a transactions tree are therefore included in the block body. The reputation of a UAV can be affected by suspicious behaviors, such as checking privacy data against access policies provided in block headers and producing or relaying incorrect blocks. The reputation value for each UAV is stored using an MPT-based cryptographically authenticated data structure, which can be shown in Figure 4. This allows us to rapidly and efficiently detect data that have changed without retrieving the entire dataset to compare.

#### 4.3.1. Transaction Detail

Transactions are defined as communications between GCS, UAVs, and cloud servers among the entire system. A microsized transaction structure, as indicated in Table 3, is recommended due to the limited storage capacity available in UAVs. A transaction's details include the requester and recipient's transaction type IDs (similar to blockchain addresses), the requester's signature (i.e., the sender), and other data required. In contrast to the addresses used in the blockchain, shorter IDs are utilized to identify UAVs, and the extra data can range in length from 0 to 1024 bits.

#### 4.3.2. Handling Transactions

Based on the distributed BC-based architecture presented in Figure 3, analysis is performed on how transactions are handled in the semiautonomous UAVs system.

1. *Genesis*: genesis defines the process of adding additional devices before commencing the mission, which should be established by GCS administrators after authentication, while each UAV is given a unique ID and a pair of public/private keys to allow it to sign transactions. Because each UAV has a starting reputation value of 69, which denotes its trustworthiness, and because the value fluctuates in response to the UAV's bad behavior.
2. *Command*: to launch a command transaction, GCS must either request data from the UAV or send a control command to UAV equipment. There are several types of information that may be included: airborne GPS data (such as altitude and speed, for example), flight data (such as acceleration and deceleration), sensor data from UAVs (such as cameras), and picture data (such as photos and films taken with the camera). Command transaction is only applicable in the case where a large number of UAVs are within a short distance of the GCS. There is a DATA field in GCS's transaction that specifies what sort of data was gathered. Command transactions are followed by time windows in which UAVs must follow through with the command. Another option is to use artificial means of capture, altering the policy list to prevent communications with that UAV, or lowering its reputation value to alert other UAVs in its network about the suspect.
3. *Inquiry*: some UAVs may require data from other UAVs following the specified policy. Some UAVs require information on the flight path of other UAVs to plan the route more accurately while modifying their respective direction. Note that the transaction may only be initiated by UAVs. It is possible that with the presented technique, the requester may have to send a resend request within a time window Δ*t*. The requester would launch a report transaction broadcasting the suspicious activity of a specific UAV if it did not receive a response in the context of a lawful query within Δ*t*.
4. *Respond*: when a request is made that breaches an access restriction, the requesting party should make a report transaction detailing the infraction and disseminate it to punish the suspect UAV to some degree. Requestors would instead respond to requests according to the policy's satisfaction level, which is determined by the reputation value of the requestee. Diffie–Hellman shared key would be used by the receiver, and the answer would be sent back to the requester. The shared key is used to send the response back to the requester.
5. *Access*: the terms “access transaction” and “UAV/GCS cloud server interaction” are used interchangeably here. Transactions should be checked against the most recent policy list by any UAVs that receive them. To punish the person who requested access, the transaction should be discarded and a report should be published.
6. *Store*: it is the cloud server's job to store data from drones and ground control stations (GCS). To verify the transaction, the cloud server checks to see whether there is enough storage space available. The hash value of the received data is then calculated and compared to the received hash value. Once two hash values match, the data packets are stored in the cloud. This is followed by encoding the requested address with the Diffie–Hellman method and sending it back to the user.
7. *Report*: system-wide self-monitoring and semiautonomy can be improved by the use of report transactions. If a hacked device is identified, either the UAV or GCS has the right to report it. Consequently, the reputation value will be lowered if the report is shown to be accurate. In circumstances of the dispute, such as the selection of a committee to mine the blocks and the acceptance or rejection of the report transaction, the transaction is meant to assure certain convergence towards a consensual conclusion. Voting in elections, for example, requires a single node to begin a vote transaction, and the other nodes answer with a response transaction that includes the candidate IDs and signatures of each node. Voting results are calculated after receiving the messages, and the new committee is announced to everyone.
8. *Alert*: all the information is identified to know about voting. There are ways to protect the system from future cyberattacks, such as having each UAV and GCS have alert transactions that sound an alarm if it detects a certain type of attack. It would help the system as a whole defend itself and limit losses early on. In response to varied attacks, all of the UAVs in the network would take the same course of action.

## 5. Working Mechanism

**Figure 5:**
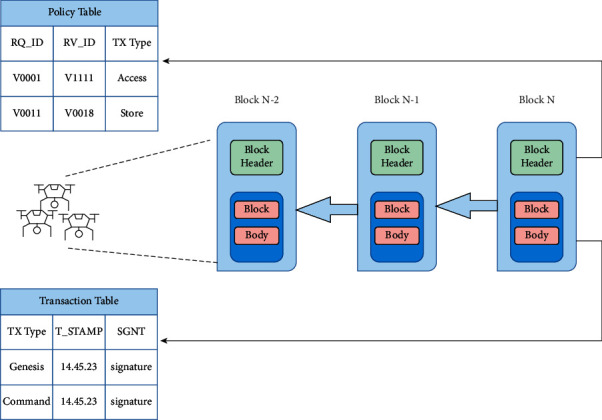
Proposed UAVs blockchain architecture.

A distributed ledger, consisting of connective blocks, is used to store all communications data in the current system. This ledger is present in both UAVs and GCS. Message transmission, message verification through a voting system, and mining all require accuracy guarantees to maintain data security. The presented framework's functioning mechanism as shown in Figure 5, including data processing, reputation evaluation, and consensus method, is explained in-depth ahead.

### 5.1. Processing of Data

#### 5.1.1. Registering a New account

To join the network, any device (such as an unmanned aerial vehicle, or UAV) must first register using the genesis transaction. Based on its MAC address, the most recent timestamp, and random salt hash value, each device generates its private key before registration. Preloaded policies for each node will also outline what actions to do while receiving messages.

#### 5.1.2. Data Hashing

Each node stores the public keys of all other nodes, its private key, and the next blocks in a sequence. Cryptography procedures like the hash function and digital signature must be performed on all devices before it delivers communications. In comparison to other lightweight hash functions (such as Quark, PHOTON, and SPONGENT), Keccak is a high-performance hash function in both code size and cycle count. Due to the extensive use of hash functions, such as block hash, prior hash, reputation root, transaction root, and the message digest for each transaction, the 160-bit output is shortened to 80-bit to conserve memory.

#### 5.1.3. Verification of the Data

If two digests match, the data integrity and consistency of a transaction in a peer-to-peer network are verified. Whether the request is genuine, the policy list is checked to see whether it satisfies all of its requirements. Reputation values of individual UAVs are reduced if a transaction is rejected due to inaccuracies in data integrity or if it violates a set of policies, which are communicated to other UAVs via a report transaction issued by receivers of rejected transactions. As a result, the reputation worth of the person who correctly reports the hostile behavior rises. For example, each report transaction would trigger a voting procedure in which each node votes on its verification result and the reputation value of the suspected UAV in a distributed voting mechanism.

### 5.2. Estimation of Reputation Measure

To confirm the validity of the received blocks, a distributed reputation assessment mechanism is adopted that reduces the block verification cost. Merkle Patricia Trie is used to hold the reputation value of all nodes in the proposed architecture. Each group of UAVs is led by a master UAV and supported by a swarm of general UAVs. Using the reputation assessment, the system keeps track of each node's trustworthiness. For the most part, each UAV starts with a reputation value of 69, which may either be boosted or lowered depending on whether or not the UAV is successfully reported as suspicious. It is also vital to note that each UAV in the network can accept or relay transactions based on its reputation value, which is calculated using the following equations:

(1) $$\begin{array}{c} \mu =1,\text{if }s>99,\\ \mu =\frac{\mu_{1}*s}{\sum_{k=1}^{O}s_{k}*D_{j}}\text{,if }59<s<99,\\ \mu =\frac{\mu_{2}*s}{\sum_{k=1}^{O}s_{k}*D_{j}}\text{,if }s<59. \end{array}$$

If UAV *V*_*j*_'s reputation value is more than 59 and its number of suspicious acts is greater than 59, *μ* reflects the likelihood that UAV *V*_*j*_'s request will be accepted. Conspicuously, a high reputation value encourages the acceptance and trustworthiness of its communications and vice versa. If the reputation value of the requester falls below 29, neighboring UAVs will refuse to transmit all of the transactions started by it. If UAVs are not programmed to transmit lots of spam or bad messages, it makes it harder for them to take over the system. To compute the reputation of a connected node, a node takes into account the quality of service provided by its peers. Coefficients *μ*_1_ and *μ*_2_ are used to weigh the relevance of connections in the current system.

#### 5.2.1. Distributed Voting System

ID-based vote distribution technique has several functions. DPoS-like distributed consensus protocol is proposed to agree with the acquired data. As a result, the committee that generates the block is elected by a vote. It is well known that a report transaction is used to alert authorities of suspicious activity by UAVs. However, the hacked UAV may undermine the system's availability by inventing report transactions to frame the conforming UAVs. As a result, the voting mechanism should assess the legitimacy of each report transaction. Moreover, the voting procedure is used to handle instances when there is a lot of disagreement. Vote transactions are used to compare the GPS of a UAV to those of other UAVs in a no-fly zone, for example, when one UAV unexpectedly finds itself in or near the no-fly zone without any anticipation. A GPS spoofing attack is extremely likely if it does not match, and the UAV should send out an alert to warn its neighbors so they can take precautions to reduce the danger. To further understand the voting process, assume a network of *O* UAVs. Each node in the network can cast a vote based on the outcome of its verification and its conclusion. If the total number of votes is less than or equal to *O*, then voting is permitted as (*L*/*O*) > *π*, where *π* denotes the threshold value. To ensure that a result is accepted by the majority of nodes in the system, the threshold *π* must be larger than 50%.

### 5.3. Consensus Protocol

In distributed and multiagent systems like UAVs, the consensus method is critical for building trust and dependability in the network. A detailed explanation of how the system works, including the rules for generating committee selection blocks, is provided in the current section.

#### 5.3.1. Selection of Committee

An ideal scenario for selection is one where GCS and UAVs are nearby, allowing for high-quality intersystem communications. Given that GCS can be trusted, it makes sense to designate GCS as the miner in charge of collecting all transactions, verifying the validity, and managing changes to reputation values in the block header, all of which relieves the UAVs from the computational burden. Instead, if the swarm of UAVs must coordinate independently on a mission without continuous connections to GCS, this technique, “Voted Nodes as Miners,” is more suited. The administrator of GCS should form a committee based on the roles assigned to UAVs during the system's infancy, and the committee's membership should be proportional to the entire number of UAVs. The reelection of the committee is triggered by any block generation or forks in the blockchain ledger that are not recorded. If this is the case, committee members are chosen based on their reputation value, with only the top 16% of nodes eligible to serve. To form the final committee, each node will vote for 3 of the top 5 candidates, and the master UAV will generate a vote transaction. The results of the voting will be shown to everyone on the whole network.

#### 5.3.2. Generation of Blocks

The accumulative transactions in a block might generate communication delays or slow down the transmission rate among the network if the block generation rate is vague. Otherwise, the blockchain system's nodes might become overburdened with processing if mining occurs too often. The presented design relies on blocks being generated at the right place; hence, a set block creation rate is recommended. Each block is generated at a certain time slot, which necessitates a regular rotation of the mining tasks. After the previous block is generated, the mining process immediately moves on to the next fresh round. Because various jobs have varying communication requirements, the time interval between two rounds of block creation may be customized. Time interval of mining, average block size, and time interval of periodic memory release are all variables that may be used to model an O-UAVs network's data storage capacity. The following restriction applies to the current work;

(2) $$\begin{array}{c} \alpha *\text{floor }\left(\frac{t_{0}}{\beta }\right). \end{array}$$

**Figure 6:**
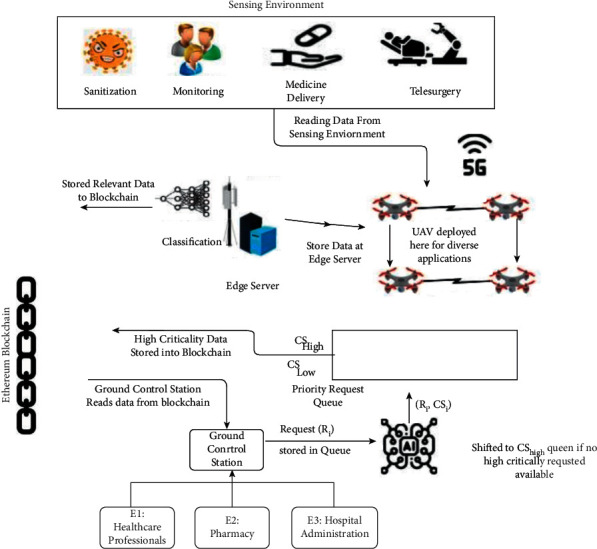
Blockchain-based intelligent surveillance: healthcare environment.

When rounding to the closest integer, floor (.) is used. Indeed, the restriction assures each device has enough memory to store data in the blockchain until its next memory release.  Figure 6 depicts the illustrative view of healthcare applications based on the presented model.

## 6. Performance Analysis

### 6.1. Security Analysis

This section details the performance analysis of the proposed model with respect to different attacks.

#### 6.1.1. Sybil Attack Protection

In the suggested architecture, harmful requests are kept away from devices to boost availability by restricting the access privileges of each network participant by policy list to those entities that hold certain vital information of the system. To avoid message transformation, transactions received from other UAVs are authenticated by each device before being sent on to the neighbors.

#### 6.1.2. Mitigation of DoS/DDoS

A DoS/DDoS attack is less likely to occur in our system because network nodes will not broadcast garbage information if the sender's reputation value falls below a certain level. The packet flows between UAVs may be monitored by GCS, and as a result, GCS might reset the policy list to block all access permits, therefore decreasing the impact of a tampered UAV.

#### 6.1.3. Resistance against GPS Spoofing

The proposed method has some resistance to GPS spoofing since the voting transaction may obtain other UAVs' GPS information. As a general rule, it is hard for an attacker to take over all of the UAVs at the same time. Once a UAV identifies itself in or near a no-fly zone, it will transmit a vote transaction to claim its GPS data. Allowing participants to respond with true/false messages would be referred to as a vote function. As a result, the requester could verify that it had the correct GPS data.

#### 6.1.4. Consensus Protocol Security

Blockchain-based UAV require high levels of security for the blockchain consensus mechanism that underpins them. Natoli et al. [50] summarized the security of consensus protocols under various attack models, including miner power attacks, strategic miner attacks, communication attacker attacks, hybrid attacker attacks combining strategic mining with communication attacks, and stake attacker attacks. As a result of the proposed consensus scheme's reputation, attacks can be prevented from both mining power and strategic mining. However, the current system is vulnerable to communication attacks, strategic mining and communication attacks, and stake attacks.

### 6.2. Evaluation of Efficacy

**Figure 7:**
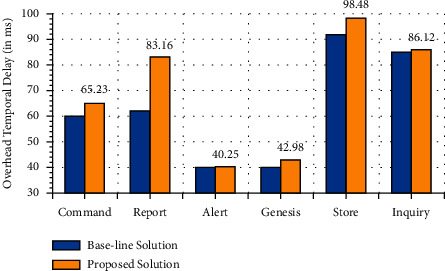
Overhead temporal delay.

**Figure 8:**
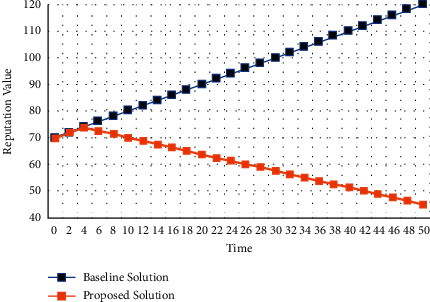
Reputation value estimation.

**Figure 9:**
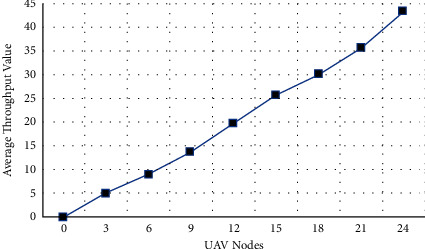
Throughput of proposed solution.

The proposed distributed BC-based technique is deployed using an emulator for multiagent UAVs networks termed as UB-ANC Emulator based on the technique of ns-3 [51]. The entire system configuration can be seen in Table 4. Moreover, the simulation parameters used in NS-3 are depicted in Table 5. The suggested architecture was compared to a base system that does not use a digital signature, hashing, or blockchain technology. The experiment excludes the average delay of transmission because of the limitations of the simulator. Figures 7–9 show the outcomes of the simulation trials. When it comes to drone networks, a novel method is presented in the current study that decreases computational and communication overhead while enhancing the security and privacy of drone systems. According to Figure 7, which shows the time overhead for various types of transactions, the most time-consuming portion is the store inquiry. Most transactions in the proposed system would take longer or cost more than they would in the current scheme. Inquiry transactions take less time in our solution because UAVs in the presented framework may choose more creditable objects to collect information, saving a lot of time waiting for important replies. Reputation values for malicious nodes have been fluctuating over time as seen in Figure 8. The reputation value of the malicious node might be viewed as rising linearly over time if the other complying nodes are unable to identify any malicious behavior in the basic scheme without the reputation evaluation scheme This is not the case in our suggested method, whereby a hacked node's reputation quickly falls below the threshold value (59). It can be seen from the graph that the reputation of a rogue node deteriorates over time. Other nodes will not trust a malicious node when its reputation falls below a certain level. As a result, the UAV swarm's distributed autonomous decision-making may be assumed to be secure. The average throughput of the proposed BC-based framework increases as the number of UAVs in the network grows. Finally, Figure 9 depicts the cumulative throughput of the proposed model in comparison to the baseline techniques. Furthermore, Table 6 depicts the packet flow evaluation for the proposed model in comparison to the proposed approach.

### 6.3. Statistical Performance

**Figure 10:**
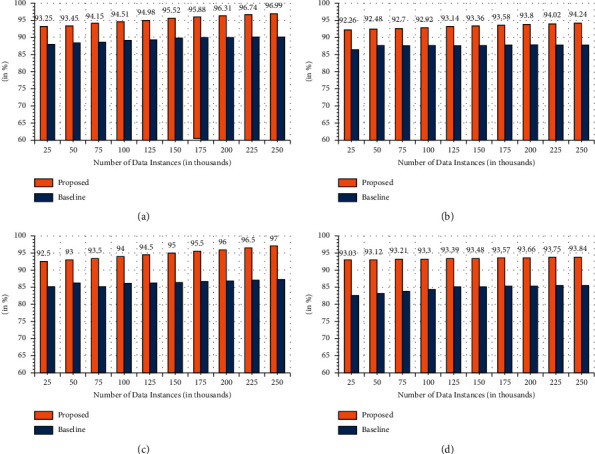
Statistical performance. (a) Precision. (b) Specificity. (c) Sensitivity. (d) *F*-measure.

In addition to the performance analysis mentioned before, the proposed model is deployed to determine the statistical performance in terms of precision, specificity, sensitivity, and f-measure. For comparative analysis, the baseline technique is used.  Figure 10 shows the overall results of the proposed technique. It can be seen that in the current scenario, the proposed technique can register an enhanced precision measure of 92.15% (Figure 10(a)) concerning 83.15% of the baseline technique. Moreover, the specificity analysis shown in Figure 10(b) shows that the presented technique acquired a better measure of 93.65% in comparison to 88.45% for baseline. Furthermore, the enhanced measure of sensitivity (Figure 10(c)) and f-measure (Figure 10(d)) are registered for the presented technique showing that the proposed technique is more effective and efficient for detecting attacks in comparison to the baseline techniques.

### 6.4. Stability and Reliability Estimation

**Figure 11:**
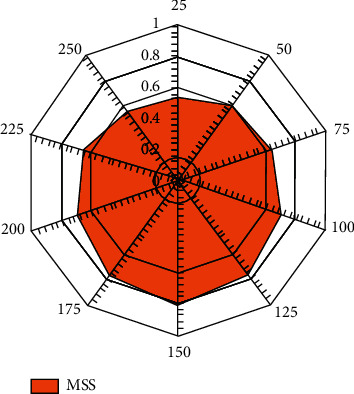
Stability analysis.

Stability refers to the normalized behavior of the proposed algorithm over the variable number of data events. In the current scenario, the stability is measured in terms of the mean stability measure (MSS), where the value of MSS lies between 0 and 1. 0 represents low stability and 1 indicates higher stability.  Figure 11 shows the overall results for the proposed security technique over the variable number of data sets. Factually, the data instances are bootstrapped to 250000 for determining optimal results.

**Figure 12:**
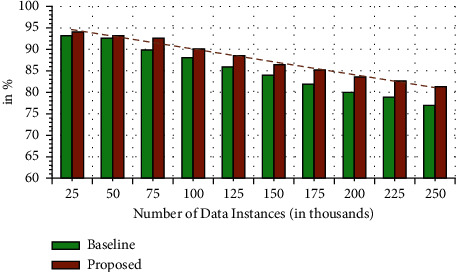
Reliability analysis.

Reliability is another vital performance parameter in the current domain of study. Specifically, it deals with the durability of the proposed technique for attacks that can be carried out. In other words, the reliability performance is mapped using the failure rate, in case any data attack is performed in the current technique.  Figure 12 shows the overall results of reliability for the proposed technique. It can be seen that in the current scenario, the proposed model can register enhanced reliability of 90.01% for different data instances in comparison to 85.26% for the baseline technique. It shows that the presented technique is more reliable for data security attacks variable attacks.

## 7. Open Issues

**Figure 13:**
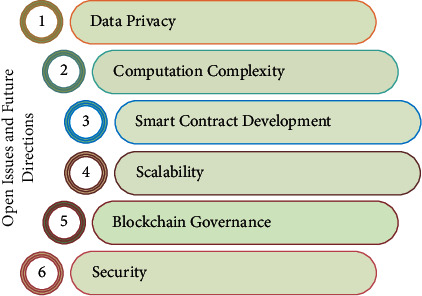
Open issues and challenges.

Blockchain, AI, and UAVs are being used in a wide range of applications, including healthcare, defense, smart cities, and the smart grid. Despite the many advantages of integrating drones with blockchain, AI, and 5G, there are still several issues that must be overcome. As seen in Figure 13, the main research problems of the suggested strategy are discussed below.

1. *Privacy*: to ensure the proposed scheme's participants' data privacy, the blockchain used in the proposed scheme is public and available to all of its participants. As a result, although data collection and storage on the blockchain increase security and improve UAV communication performance, sensitive information about users is also available. This raises concerns about the privacy of blockchain data, and corporations are limiting the use of the blockchain as a result. There is a great deal of demand for blockchain privacy-preserving technologies.
2. *Difficulty of computation*: UAV communication security and network performance will be improved with the proposed system's usage of AI and 5G as an intermittent technology. Here, 5G enables tremendous data speeds and ultralow latency, resulting in massive amounts of data being generated. With so much data being created, the edge-AI algorithm (limited space and compute capacity) cannot manage it.
3. *Development of smart contracts* because smart contracts are immutable, it forces programmers to take extra care when creating them. The blockchain network can be severely damaged if a smart contract has a flaw or software vulnerability. To ensure the safety and security of the blockchain network, engineers must conduct a security and vulnerability evaluation.
4. *Ability to scale up* for the blockchain-aided UAV network, this is one of the most pressing issues to address. A blockchain conducts an average of 12 transactions per second, which is too slow for a 5G network. Blockchain's performance is appalling in comparison to social media platforms like Facebook, Instagram, and Twitter, which process millions of transactions every second. Because of this, the blockchain network must be made more scalable.
5. *Chain of command* in a public blockchain network that is completely decentralized and distributed, deploying and managing a distributed ledger (shared by several participants) is a difficult undertaking. Issues such as who oversees, manages, and troubleshoots the blockchain network emerge even in private/consortium blockchain. Other concerns include who is responsible for creating and deploying smart contracts, how disputes are resolved, and the rules and norms that govern blockchain. A strong and efficient blockchain governance paradigm is therefore required.
6. *Protection* using public-key cryptography, a blockchain network's data are protected from unauthorized access (digital signatures). Quantum computing, a futuristic notion, has the potential to decrypt public key encryption. By 2027, researchers predict that quantum computing will be able to compromise the blockchain's security. As a result, a quantum-secure blockchain network is required.
7. *Delay in data processing* many different sensors on UAVs can create a vast quantity of data, which can lead to suboptimal solutions due to the battery constraints of UAVs (not entirely correct).
8. *Standardization of the blockchain* companies have yet to settle on how to use blockchain technology. As a result, the establishment of a real-time UAV network may impede its use.

## 8. Conclusion

IoT formulates a significant domain for provisioning real-time services in UAV applications. In the current research, security and privacy risks associated with the UAV system are addressed using a novel blockchain technique. With the proposed architecture, UAV-based applications can collect sensed data via a trustworthy platform. Specifically, the proposed framework is designed to remove storage constraints in the IoT environment. Moreover, a novel consensus algorithm is proposed with a reputation assessment system. Conspicuously, data gathering in real-time ensures integrity, confidentiality, and availability. Finally, a working prototype is proposed based on the proposed UAV system and tested in a real-world environment for performance enhancement. Based on the results, optimal results were registered in terms of statistical parameters of temporal efficacy, stability, reliability, and security analysis. For future works, research can be performed on communication-based data security. Moreover, network drop-constraint is another direction of research in the current domain.

## Figures and Tables

**Table 1:** Comparative assessment (YY: available, NA: not available).

Reference	Major contribution	Blockchain	M2M communication	Cognitive decision	Security	Real-time	Performance analysis	Numerical quantification	Packet evaluation	Statistical analysis	Temporal delay
Alemany et al. [22]	Blockchain-based connectivity provisioning	YY	YY	NA	NA	YY	YY	YY	NA	NA	NA
Singh et al. [23]	Deep-learning-based blockchain framework for secure industrial networks	YY	YY	YY	YY	NA	YY	NA	NA	NA	YY
Jamal et al. [24]	Lightweight and scalable control plane management	YY	NA	NA	YY	NA	YY	NA	YY	NA	NA
Bagci et al. [25]	Dynamic QoS-path optimization in multioperator services	YY	NA	NA	YY	YY	YY	YY	NA	NA	NA
Fernando et al. [26]	Blockchain-powered software defined networking infrastructure	YY	YY	NA	YY	YY	YY	NA	YY	NA	NA
Chattaraj et al. [27]	Design of blockchain-based access control scheme	YY	NA	YY	NA	NA	NA	YY	NA	NA	NA
Do et al. [28]	Joint optimization of real-time resource allocation for UAV	NA	YY	YY	NA	NA	YY	NA	NA	YY	NA
Xiao et al. [29]	Modeling and verifying SDN under multicontroller architectures	NA	YY	NA	NA	YY	YY	NA	YY	NA	NA
Yao et al. [30]	Resource allocation for 5G-UAV	YY	YY	NA	YY	NA	NA	NA	NA	NA	NA
Proposed technique	Distributed blockchain-based platform for unmanned aerial vehicles	YY	YY	YY	YY	YY	YY	YY	YY	YY	YY

**Table 2:** Block composition.

Contents	Description	Size (bits)
T_STAMP	Linux timestamp of the block	32
B_HASH	Hash value of current block header	78
P_LIST	Access rules of a fleet of O UAVs	30 *∗* O
T_ROOT	Root of the transaction tree	78
P_HASH	Hash value of previous block header	78
R_ROOT	Root of the reputation tree	78

**Table 3:** Transaction composition.

Contents	Description	Size (bit)
T_TYPE	Transaction type	8
RV_ID	Device ID of the receiver	16
RQ_ID	Device ID of the sender	16
DATA	Additional information	1024
SIGNATURE	Signature/multi-signature	1024/2048

**Table 4:** Simulation settings.

Operating platform	64-bit Linux
CPU	2.57 GHz intel i7 core
Network simulation tool	ns3
Ground control station tool	QGround-control

**Table 5:** Simulation parameters.

Simulation parameter	Measure
Radio link control (RLC) buffer size	150
Pkt size	998 bytes
Transmission control protocol (TCP) traffic type	Cubic
Application data rate	98 Mbps
Movement speed	18 m/s
Simulation area	6 km radius
Number of traffic source	8
Wired link delay	48 ms
Number of resource block	18
Mobility	Random walk 2D
Wired link capacity	8 Mbps

**Table 6:** Packet flow evaluation.

Packet flow	Proposed	Baseline
From UAV to UAV	29	14
From UAV to cloud	39	19
From UAV to GCS	37	19
From GCS to cloud	63	59

## Data Availability

The data that support the findings of this study are available on request from the corresponding author.
